# Left ventricle- and skeletal muscle-derived fibroblasts exhibit a differential inflammatory and metabolic responsiveness to interleukin-6

**DOI:** 10.3389/fimmu.2022.947267

**Published:** 2022-07-28

**Authors:** Isabell Matz, Kathleen Pappritz, Jochen Springer, Sophie Van Linthout

**Affiliations:** ^1^ Berlin Institute of Health at Charité - Universitätmedizin Berlin, Berlin Institute of Health (BIH) Center for Regenerative Therapies (BCRT), Berlin, Germany; ^2^ German Center for Cardiovascular Research (DZHK), Partner Site Berlin, Berlin, Germany

**Keywords:** fibroblasts, left ventricle, skeletal muscle, IL-6, NLRP3 inflammasome

## Abstract

Interleukin-6 (IL-6) is an important player in chronic inflammation associated with heart failure and tumor-induced cachexia. Fibroblasts are salient mediators of both inflammation and fibrosis. Whereas the general outcome of IL-6 on the heart’s function and muscle wasting has been intensively studied, the influence of IL-6 on fibroblasts of the heart and skeletal muscle (SM) has not been analyzed so far. We illustrate that SM-derived fibroblasts exhibit higher basal mRNA expression of α-SMA, extracellular matrix molecules (collagen1a1/3a1/5a1), and chemokines (CCL2, CCL7, and CX3CL1) as compared to the left ventricle (LV)-derived fibroblasts. IL-6 drives the transdifferentiation of fibroblasts into myofibroblasts as indicated by an increase in α-SMA expression and upregulates NLRP3 inflammasome activity in both LV- and SM-derived fibroblasts. IL-6 increases the release of CCL7 to CX3CL1 in the supernatant of SM-derived fibroblasts associated with the attraction of more pro(Ly6C^hi^) versus anti(Ly6C^lo^) inflammatory monocytes as compared to unstimulated fibroblasts. IL-6-stimulated LV-derived fibroblasts attract less Ly6C^hi^ to Ly6C^lo^ monocytes compared to IL-6-stimulated SM-derived fibroblasts. In addition, SM-derived fibroblasts have a higher mitochondrial energy turnover and lower glycolytic activity versus LV-derived fibroblasts under basal and IL-6 conditions. In conclusion, IL-6 modulates the inflammatory and metabolic phenotype of LV- and SM-originated fibroblasts.

## Introduction

Heart failure (HF) and cancer are both main causes of morbidity and mortality worldwide (1). Increasing evidence suggests a multifaceted relationship between both disease entities. The relevance of inflammation as a trigger and valuable therapeutic target in HF and cancer was shown in the CANTOS (Canakinumab Anti-Inflammatory Thrombosis Outcome Study) trial. Beyond the primary observation that rates of cardiovascular events were lower in canakinumab-treated patients with previous myocardial infarction as compared to the placebo group (2), the largest cytokine inhibition trial ever performed illustrated that interleukin (IL)-1β antagonism reduced the incidence of lung cancer and cancer-related mortality (3). Beyond being a common contributor to the pathogenesis of cancer and HF, inflammation associated with cancer can provoke HF (4). In addition, there is further recognition that systemic inflammation associated with HF (5) stimulates tumorigenesis (6). Furthermore, in both disease entities, chronic inflammation can lead to cachexia, a metabolic syndrome that is characterized by high morbidity and mortality due to involuntary weight loss caused by the depletion of fat mass and a prominent loss of muscle tissue (7) leading to a severely impaired muscle function. Less known is the evidence from experimental (8–10) and patient (11) studies illustrating that HF-elevated serum cytokine levels are associated with increased local inflammation in the skeletal muscle (SM).

One of the inflammatory factors secreted from various cancers known to heavily impact cachexia, and which is stimulated, *inter alia*, by IL-1, is IL-6 (12–14). Blocking IL-6 signaling leads to decreased wasting of muscles and prevents cancer cachexia in melanoma and prostate tumor cachectic mouse models (15). Permanent excess of IL-6 during chronic inflammation exerts malignant effects on cardiac function (16), whereas IL-6 spillover in the peripheral circulation increases with the severity of HF, and enhanced IL-6 levels are associated with reduced clinical outcomes (17, 18). IL-6 gene expression is elevated in the heart of tumor-bearing mice, which are characterized by impaired cardiac function and increased fibrosis (19), hinting to the possible involvement of cardiac fibroblasts in tumor-induced cardiac dysfunction in rodents and humans (20, 21).

In addition to their function of extracellular matrix (ECM) regulation, fibroblasts gain more and more recognition for their role as inflammatory supporter cells in the heart (22–26). Fibroblasts present a heterogeneous cell population, located at different sites of the body, and are of mesenchymal origin (27). Recent single-cell analysis of fibroblasts from the heart and SM illustrated fibroblast heterogeneity and an organ-specific fibroblast-mediated ECM profile (28), although how fibroblasts from the left ventricle (LV) versus SM may exhibit an organ-specific response to IL-6 has not been analyzed before.

Although the overall effect of IL-6 on cardiac function (16–19) and SM function/wasting (15) has been deeply investigated, little is known so far about the effect of IL-6 on cardiac and SM fibroblasts. As fibroblasts are one of the main drivers of inflammation (22–26), including the main source of NLRP3 inflammasome activity (29) in the heart, and their role as inflammatory supporter cells is underexplored in the SM, this work aimed to elucidate the impact of IL-6 on both LV- and SM-derived fibroblasts. These investigations provide further insights into how fibroblasts from different organs may respond differently toward a common systemic inflammatory response (here IL-6) and can support local and potentially chronic systemic inflammation.

## Methods

### Animal material

LV and SM tissues were isolated from 12-week-old healthy male C57BL6/j mice (n = 6; Charles River, Sulzfeld, Germany). All animals were housed under standard housing conditions (12-h light/dark cycle, 50%–70% humidity, 19°C–21°C) with free access to food and water. The experiments were performed in accordance with the European Directive 2010/63/EU and were approved by the local ethical committee (Landesamt für Gesundheit und Soziales, Berlin, T0025/15).

### Outgrowth culture of murine left ventricle- and skeletal muscle-derived fibroblasts

In order to isolate and expand fibroblasts from the LV and SM, an outgrowth culture (22) was performed. Therefore, LV and SM tissues were cut into 1-mm-sized pieces and were subsequently fixed in 12-well plates by scratching the pieces in the plastic of the bottom of the plates. During outgrowth, the fibroblasts were cultured in Dulbecco’s modified Eagle medium (DMEM; Gibco, Thermo Fisher Scientific Inc., Waltham, MA, USA) containing 20% fetal bovine serum (FBS) (Bio&Sell GmbH, Feucht, Germany) and 1% penicillin and streptomycin (P/S) (Gibco) at 37°C and 5% CO_2_. Stimulation experiments were performed between passages 4 and 9.

### Immunofluorescence staining of left ventricle- and skeletal muscle-derived fibroblasts

To characterize the outgrowth cells of LV and SM tissues, outgrowth cells in passage 1 were stained with markers of mesenchymal cells, cardiomyocytes, and endothelial cells, as previously described (30), using murine C4 fibroblasts (Sigma Aldrich, Munich, Germany), HL-1 cells (cardiomyocytes), and brain-derived endothelial cells (bEnd.3 cells) (ATCC, Tell City, IN, USA) as reference cells (**Supplementary Figure 1**). In detail, each cell type was seeded at a density of 10,000 cells/well in a 48-well format. Upon reaching 80% confluence, the cells were washed once with DPBS (Gibco) and subsequently fixed in 4% paraformaldehyde (PFA) (Sigma Aldrich) for 10 min and stored at 4°C until staining. For this, the cells were first permeabilized with Triton X-100 for 5 min, followed by 30 min incubation with avidin blocking solution (Vector Laboratories, California, USA). Subsequently, the cells were incubated with the primary antibody, all diluted at 1:50, for 1 h at room temperature (RT). The following primary antibodies were used: anti-vimentin (GTX 100619, GeneTex, distributed *via* Biozol, Echingen, Germany), anti-desmin (SC-7559, Santa Cruz, Heidelberg, Germany), and anti-CD31 antibody (BD550274, BD Biosciences, Heidelberg, Germany). After washing two times with DPBS (Gibco), staining with a 1:250 diluted biotinylated secondary antibody (Dianova, Hamburg, Germany) was performed. According to our established protocol, cells were finally incubated with Cy3-conjugated streptavidin diluted at 1:250 (Jackson ImmunoResearch Laboratories Inc., West Grove, PA, USA). For nuclear staining, the cells were incubated with 1:100 diluted diamidinophenylindol (Invitrogen, Darmstadt, Germany). Pictures were acquired using an Axio Observer.Z1 (Carl Zeiss Microscopy GmbH, Oberkochen, Germany) at a magnification of ×200. The microscopic images were taken and processed with the Axio Vision SE64 software (version 4.9.1, Carl Zeiss Microscopy GmbH).

### Stimulation experiments of left ventricle- and skeletal muscle-derived fibroblasts

For stimulation experiments, murine fibroblasts were seeded in 6-well plates (50,000 cells/well) for collection of supernatant and flow cytometry, in 48-well plates (20,000 cells/well) for RNA isolation, in 96-well plates (7,500 cells/well) for Crystal Violet and Sirius Red assay, or in Seahorse 96-well Utility plates (Agilent Technologies, Santa Clara, CA, USA) (20,000 cells/well) for analysis of mitochondrial function and glycolysis. The number of biological and technical replicates for each experiment is indicated in the legends of the figures. When the cells reached at least 80% confluency, the medium was replaced, after washing with DPBS (Gibco), by a DMEM containing 5% FBS and 1% P/S (basal medium). The cells were next cultured under basal conditions for 3 h. In the following step, the basal medium was removed, and the cells were incubated with either fresh basal medium (no stimulation) or DMEM including 5% FBS, 1% P/S, and 10 ng/ml of IL-6 (31) (PeproTech, Rocky Hill, NJ, USA) for up to 72 h.

### RNA isolation

For RNA isolation, the fibroblasts were harvested after 24-h stimulation with 0 or 10 ng/ml of IL-6, and the TRIzol^®^ (Life Technologies, Carlsbad, CA, USA) method was used. Following the addition of chloroform and subsequent centrifugation, the upper aqueous phase, containing the RNA, was removed and precipitated with isopropanol (Carl Roth GmbH, Karlsruhe, Germany). The RNA was washed in 70% ethanol, and the pellet was next dissolved in 10 μl of RNase-free water (Invitrogen).

### cDNA synthesis and real-time PCR

After DNase treatment (DNAse Kit, Peqlab, VWR International, Radnor, PA, USA) and the addition of an RNase Inhibitor (Promega, Fitchburg, WI, USA), 1,000 ng of RNA was transcribed into complementary DNA (cDNA) using the High-Capacity Reverse Transcriptase Kit (Applied Biosystems, Darmstadt, Germany). To assess the mRNA expression of the target genes, real-time PCR was performed using the Quant Studio 6 Flex TaqMan system (Applied Biosystems).The following commercially available gene expression assays (all Applied Biosystems) were used: alpha-smooth muscle actin (α-SMA; Mm00725412_s1), collagen 1a1 (Col1a1; Mm01302043_g1), collagen 3a1 (Col3a1; Mm00802331_m1), collagen 5a1 (Col5a1; Mm00489299_m1), glyceraldehyde 3-phosphate dehydrogenase (GAPDH; Mm99999915_g1), lysyl oxidase 1 (LOX1; Mm00495386_m1), lysyl oxidase-like 2 (LOXL2; Mm00804740_m1), C-C Motif Chemokine Ligand 2 (CCL2; Mm00441242_m1), C-C Motif Chemokine Ligand 7 (CCL7; Mm00443113_m1), C-X3-C Motif Chemokine Ligand 1 (CX3CL1; Mm00436454_m1), and interleukin-6 receptor (IL6R; Mm01211445_m1). For quantification of gene expression, quantitative real-time PCR data were acquired using the QuantStudio software (version 1.2, Thermo Fisher Scientific). For the expression analysis under basal conditions, the target genes were normalized to the GAPDH housekeeping gene and expressed as 2^−ΔCT^. For the stimulation experiments, data were expressed as 2^−ΔΔCT^, reflecting normalization of the target genes toward GAPDH and subsequent normalization toward the mean of the basal conditions of each tissue.

### Crystal Violet and Sirius Red assay

To assess the total collagen content per well of (un)stimulated LV- and SM-derived fibroblasts, a Sirius Red assay was performed 24 h after stimulation with or without (basal condition) 10 ng/ml of IL-6 as previously described (32, 33). The cells were fixed in 100 µl of methanol (Thermo Fisher Scientific) per well and stored at −20°C overnight. After washing with DPBS (Gibco), 100 µl 25% Direct Red 80 solution was added per well and incubated for 60 min at RT. The cells were three times washed with 0.1% acetic acid solution (Thermo Fisher Scientific). To elude the staining solution, 100 µl of 0.1 N of natriumhydroxide was added, and the cells were incubated for 1 h at RT while shaking. The absorbance was measured at 540 nm using a spectrophotometer (SpectraMax 340 PC, Molecular Devices, San Jose, CA, USA), and the spectroscopic data were acquired using the SoftMax^®^7Pro software (Molecular Devices).

In order to be able to normalize the total collagen content to the amount of cells/well, a Crystal Violet assay was performed according to the same cell culture conditions as the Sirius Red assay. The cells were fixed in 100 µl of 4% PFA per well, and the cells were stored at 4°C overnight. Upon washing with bi-distilled water, 50 µl of Crystal Violet solution (Sigma Aldrich) was added per well. After 30 min of incubation at RT, the cells were washed three times with bi-distilled water. Next, 100 µl of a 1% sodium dodecyl sulfate (Sigma Aldrich) solution was added, and the cells were incubated for 1 h at RT while shaking to dissolve the Crystal Violet. The absorbance was measured at 595 nm as written above.

### CCL2, CCL7, Cx3CL1, and gp130 ELISA

To determine the amounts of secreted CCL2, CCL7, and Cx3CL1 chemokines (14) from (un)stimulated LV- and SM-derived fibroblasts, 72 h post-stimulation with 0 or 10 ng/ml of IL-6, the murine MCP-1 (CCL2) standard ABTS ELISA Development Kit and murine MCP-3 (CCL7) Standard ABTS ELISA Development Kit (both PeproTech), and the murine Cx3CL1 ELISA kit (Abcam, Cambridge, UK) were used according to the manufacturer’s protocol, respectively. For analysis of glycoprotein (gp)130 secretion in the supernatant of (un)stimulated LV- and SM-derived fibroblasts, the mouse gp130 ELISA kit (Abcam) was used as indicated by the manufacturer. To enable normalization of CCL2, CCL7, Cx3CL1, and gp130 to the protein concentration of the supernatant, a bicinchoninic acid (BCA) assay (Pierce™ BCA™ Protein-Assay, Thermo Fisher Scientific) was performed according to the manufacturer’s protocol.

### CytoSelect™ cell migration assay

To assess the chemotactic potential of fibroblast’s secreted factors, a cell migration assay (Cell Biolabs, San Diego, CA, USA) was performed as described by Pappritz et al. (2018) (14). In detail, 10^5^ splenocytes isolated from 9–12-week-old healthy male C57BL6/j mice (Charles River, Sulzfeld, Germany) were plated per well in 100 μl of RPMI 1640 medium containing 0.01% FBS and placed into the migration chamber. In the next step, 150 μl of pooled supernatant of (un)stimulated LV- or SM-derived fibroblasts from three mice was added to the feeder tray (n = 7 per condition). After incubation for 24 h at 37°C and 5% CO_2_, the migrated cells were harvested, pooled (7 wells per condition), and analyzed *via* flow cytometry staining for CD11b, CD115, and Ly6C (see below).

### Flow cytometry

Flow cytometry experiments were conducted in order to assess protein expression of α-SMA and NLRP3 inflammasome components including NLRP3, caspase-1, and IL-1β in fibroblasts. LV- and SM-derived fibroblasts were harvested after 24-h stimulation with 10 ng/ml of IL-6, resuspended in Fixation/Permeabilization solution (BD Cytofix/Cytoperm™, BD Biosciences) followed by incubation for 20 min at 4°C, and washed in BD Perm/Wash™ buffer (BD Biosciences). After an additional centrifuge step was performed and the supernatant was removed, the fibroblasts were resuspended in 100 μl of BD Perm/Wash™ buffer (BD Biosciences) containing the respective antibody conjugate diluted at a ratio of 1:100. The following antibody conjugates were used for flow cytometry: anti-α-SMA Phycoerythrin and anti-NLRP3 AlexaFluor647 (both R&D Systems, Minneapolis, MN, USA), anti-IL-1β Pacific Blue (BioLegend, San Diego, CA, USA), and anti-caspase-1 FITC (Bioss, Woburn, MA, USA). After incubation for 30 min at 18°C to 24°C in the dark, cells were washed with BD Perm/Wash™ buffer (BD Biosciences) and next resuspended in 100 μl of DPBS (Gibco) for subsequent analysis using the MACSQuant Erato (Miltenyi Biotech GmbH, Bergisch Gladbach, Germany) flow cytometer.

Migrated splenocytes were analyzed by staining of CD11b (anti-CD11b Alexa488, diluted 1:100), CD115 (anti-CD115 PerCP/Cy5.5, diluted 1:100), and Ly6C (anti-Ly6C Brilliant Violet 421, diluted 1:50) (all BioLegend). The flow cytometry data were collected using the MACSQuantify software (version 2.6, Miltenyi Biotech). Appropriate gating (**Supplementary Figures 2–4**) and analysis of flow cytometry data were performed using the FlowJo software (version 8.7, BD Life Sciences, Franklin Lakes, NJ, USA).

### Mitochondrial and glycolytic stress test

To analyze the metabolic activity of LV- and SM-derived fibroblasts after 4-h stimulation with 10 ng of IL-6, mitochondrial and glycolytic stress tests using the Seahorse XF Cell Mito Stress test kit and Seahorse XF Glycolysis Stress test kit (both Agilent Technologies) were applied, respectively. The kits were used according to the manufacturer’s protocol. In brief, the sensor cartridge was hydrated with 200 µl of Seahorse XF Calibrant (Agilent Technologies) pipetted into each well of the utility plate and incubated overnight at 37°C and 5% CO_2_. The next day, the assay medium for the mitochondrial stress test (pre-warmed at 37°C) was prepared, comprising DMEM XF Base medium (Agilent Technologies), 25 mM of d-(+)-glucose (Sigma Aldrich), 1 mM of sodium pyruvate (Gibco), and 2 mM of l-glutamine (Biochrom GmbH, Berlin, Germany). The assay medium for the glycolytic stress test consisted of 2 mM of l-glutamine (Biochrom GmbH). As assay reagents for the mitochondrial stress test, 2 µM of oligomycin (Sigma Aldrich), 1 µm of FCCP (Sigma Aldrich), 0.5 µM of rotenone (Sigma Aldrich), or 0.5 µM of antimycin (Sigma Aldrich) was added to the assay media. For the glycolytic stress test, 10 mM of d-(+)-glucose (Sigma Aldrich), 2 µM of oligomycin (Sigma Aldrich), and 50 mM of 2-deoxy-glucose (Roth) were used. Next, 25 µl of each media was pipetted into the respective port of the sensor cartridge. The cell culture medium in the utility plate was replaced by the respective assay medium. The plate was incubated under non-CO_2_ conditions at 37°C for 10 min. After the initial calibration of the sensor plate, the utility plate was placed on the tray to start the Seahorse run using the XFe96 Extracellular Flux Analyzer (Agilent Technologies). The data for Seahorse experiments were recorded and evaluated by applying Seahorse Wave Desktop Software (version 2.6.1, Agilent Technologies). After the assay was finished, the supernatant was removed from each well, and the plate was frozen at −20°C for subsequent measurement of the protein content using the Micro BCA™ Protein-Assay Kit (Thermo Scientific), which was applied according to the manufacturer’s protocol.

### Statistical analysis

Data analysis and graphical presentation were performed using the software Prism 8 (version 8.4.3, GraphPad Software, La Jolla, CA, USA). The data were presented as mean ± 95% confidence interval (CI). All the data were tested for normal distribution by using the Shapiro–Wilk test. To assess statistical differences between multiple groups, a one-way ANOVA test or the Kruskal–Wallis test with *post-hoc* Benjamini–Hochberg correction was used. Statistical difference between the two groups was assessed using the Mann–Whitney U test or Welch test. Differences between groups are presented to be statistically different with an (adjusted) p-value smaller than 0.05.

## Results

### Outgrowth culture from left ventricle and skeletal muscle tissues generates primary fibroblasts

The outgrowth cells from LV and SM tissues were characterized according to their expression of the mesenchymal marker vimentin, and the markers desmin and CD31 were able to discriminate the generated primary cells from cardiomyocytes and endothelial cells, respectively, as described previously (30). LV- and SM-derived cells were positive for vimentin but negative for desmin and the endothelial cell marker CD31. In parallel, C4 fibroblasts, serving as a positive control, showed the same signal pattern as compared to LV- and SM-derived fibroblasts. The cardiomyocyte and endothelial cell lines, which served as negative controls, were positive for their cell-specific markers desmin and CD31, respectively (**Supplementary Figure 1**).

### Skeletal muscle-derived fibroblasts exhibit a higher gene expression of α-SMA, components of the extracellular matrix, and chemokines compared to left ventricle-derived fibroblasts

**Figure 1:**
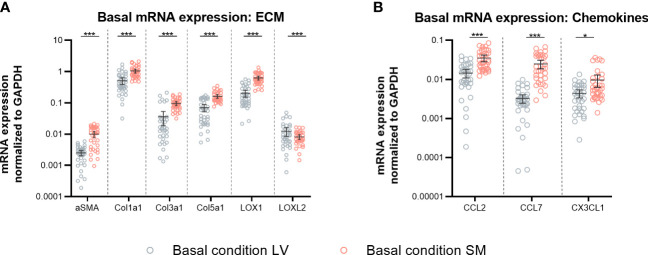
Skeletal muscle (SM)-derived fibroblasts show a higher gene expression of α-SMA, components of the extracellular matrix (ECM), and chemokines compared to left ventricle (LV)-derived fibroblasts. mRNA expression of **(A)** α-SMA, ECM components, and modulators (Col1a1, Col3a1, Col5a1, LOX, and LOXL2) and **(B)** chemokines (CCL2, CCL7, Cx3CL1) normalized to GAPDH of LV-derived (black) and SM-derived (red) fibroblasts after 24-h culture with basal medium without IL-6 (basal condition; bright hollow circle). Mean ± 95% CI; n = 36, N = 6; Mann–Whitney/Welch’s test with *post-hoc* Benjamini–Hochberg correction; adjusted p-value: *p < 0.05,***p < 0.001.

In view of evaluating potential differences between fibroblasts from the LV and SM, basal mRNA expression of α-SMA, ECM components, and modulators as well as chemokines was analyzed from fibroblasts that originated from the two different tissues. Under basal conditions, fibroblasts from the SM showed 3.9-fold (p < 0.0001), 2.0-fold, (p < 0.0001), 2.7-fold (p < 0.0001), 2.3-fold (p < 0.0001), and 3.1-fold (p < 0.0001) higher mRNA expression of α-SMA, collagen1a1, collagen3a1, collagen5a1, and LOX1 compared to LV-derived fibroblasts (**Figure 1A**), respectively. Relative to fibroblasts from the LV, SM-derived fibroblasts revealed higher mRNA expression of the chemokines CCL2 (2.4-fold, p < 0.0001), CCL7 (7.6-fold, p < 0.0001), and Cx3CL1 (2.2-fold, p = 0.0132) (**Figure 1B**). These results hint at a higher basal activity with regard to ECM modulation as well as monocyte attraction in unstimulated SM- versus LV-derived fibroblasts.

### IL-6 stimulation leads to transdifferentiation of left ventricle- and skeletal muscle-derived fibroblasts to myofibroblasts

**Figure 2:**
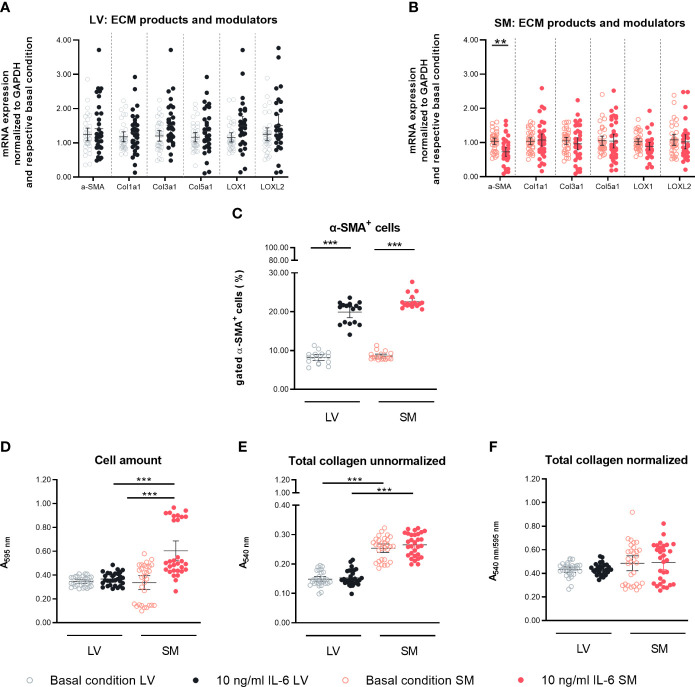
IL-6 stimulation leads to differential regulation of α-SMA in left ventricle (LV)- and skeletal muscle (SM)-derived fibroblasts. mRNA expression of α-SMA, ECM components, and modulators (Col1a1, Col3a1, Col5a1, LOX, and LOXL2) normalized to GAPDH and the respective basal condition of **(A)** LV-derived (black) and **(B)** SM-derived (red) fibroblasts after 24-h culture with basal medium without IL-6 (basal condition; bright hollow circle) or with 10 ng/ml of IL-6 (dark filled circle); n = 36, N = 6, Mann–Whitney/Welch’s test. **(C)** Percentage of gated α-SMA^+^ cells (flow cytometry data) of LV-derived (black) and SM-derived (red) fibroblasts after 24-h culture with basal medium without IL-6 (basal condition; bright hollow circle) or with 10 ng/ml of IL-6 (dark filled circle); n = 18, N = 3, Kruskal–Wallis/one-way ANOVA. **(D)** Cell number assessed *via* Crystal Violet assay and absorption at 595 nm from LV-derived (black) and SM-derived (red) fibroblasts after 24-h culture with basal medium without IL-6 (basal condition; bright hollow circle) or with 10 ng/ml of IL-6 (dark filled circle). Total collagen content of LV-derived (black) and SM-derived (red) fibroblasts after 24-h culture with basal medium without IL-6 (basal condition; bright hollow circle) or with 10 ng/ml of IL-6 (dark filled circle) measured *via* Sirius Red assay and absorption at 540 nm **(E)** unnormalized and **(F)** normalized to cell number (Crystal Violet assay data); n = 30, N = 3, Kruskal–Wallis/one-way ANOVA; all data are presented as mean ± 95% CI and tested with *post-hoc* Benjamini–Hochberg correction; adjusted p-values: **p < 0.01, ***p < 0.001.

To assess the effect of IL-6 stimulation in LV- and SM-derived fibroblasts on their transdifferentiation into (myo)fibroblasts, α-SMA gene and protein expression were analyzed (34). In addition, the impact of IL-6 on ECM components and modulators of LV- and SM-derived fibroblasts was determined. IL-6 did not affect α-SMA mRNA expression in LV-derived fibroblasts (**Figure 2A**). In SM-derived fibroblasts, IL-6 stimulation for 24 h led to the reduction of α-SMA on mRNA level compared to basal conditions (0.7-fold, p = 0.0007) (**Figure 2B**). We observed no changes in mRNA expression of components of the ECM upon IL-6 stimulation, in neither LV- nor SM-derived fibroblasts (**Figures 2A, B**). At the protein level, 24-h IL-6 stimulation induced an increase of α-SMA-positive cells in both LV- and SM-derived fibroblasts (2.4-fold and 2.6-fold, p < 0.0001) versus respective unstimulated fibroblasts (**Figure 2C**). However, a difference in α-SMA^+^ cells upon IL-6 stimulation between fibroblasts from both tissues was not observed. IL-6 increased the number of SM-derived fibroblasts (p < 0.0001), depicted as Crystal Violet-stained cells, whereas no difference in cell count was seen following IL-6 stimulation of LV-derived fibroblasts (p < 0.0001) (**Figure 2D**). IL-6 did not affect collagen deposition, in neither LV- nor SM-derived fibroblasts (**Figures 2E, F**).

### IL-6 stimulation leads to the attraction of different monocyte subsets to left ventricle- versus skeletal muscle-derived fibroblasts

**Figure 3:**
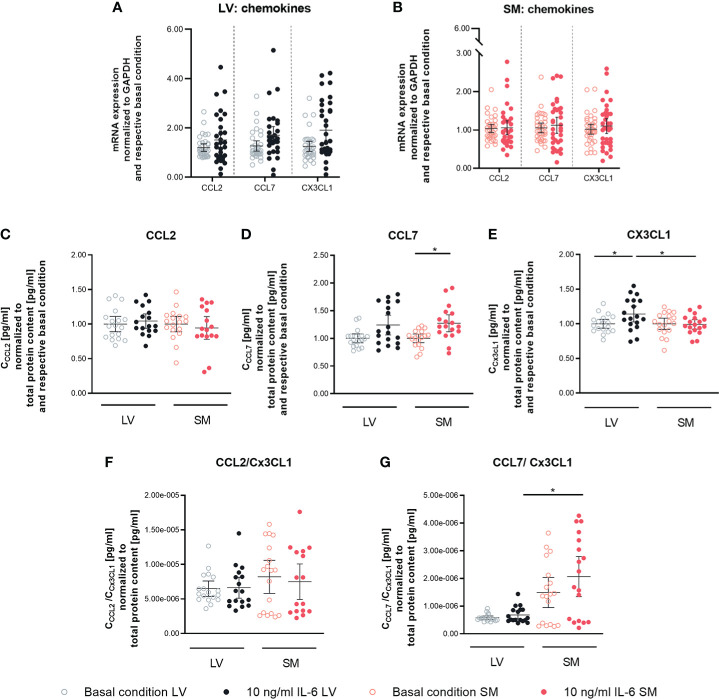
IL-6 stimulation leads to secretion of anti- and pro-inflammatory chemokines in left ventricle- and skeletal muscle-derived fibroblasts. mRNA expression of CCL2, CCL7 and CX3CL1 normalized to GAPDH and the respective basal condition of **(A)** LV-(black) and **(B)** SM- (red) derived fibroblasts after 24 h culture with basal medium without IL-6 (basal condition; hollow circle) or with10 ng/ml IL-6 (filled circle); n=36, N=6, Mann-Whitney/Welch’s test; Protein expression of **(C)** CCL2, **(D)** CCL7 and (E) CX3CL1 in the supernatant and ratio of **(F)** CCL2 and CX3CL1 and **(G)** CCL7 and CX3CL1 normalized to total protein content measured via bicinchoninic acid assay and respective basal condition of LV (black)- and SM (red)-derived fibroblasts after 72 h culture with basal medium without IL-6 (basal condition; bright hollow circle) or with 10 ng/ml IL-6 (dark filled circle); n=24, N=4, Kruskal-Wallis/One-Way ANOVA; All data are presented as mean ± 95 % CI and tested with post hoc Benjamini-Hochberg correction; adjusted p-values: *p < 0.05.

To determine the capacity of IL-6 to stimulate LV- and SM-derived fibroblasts to attract monocytes, the expression of different chemokines in LV- and SM-derived fibroblasts following IL-6 stimulation was analyzed. An impact of IL-6 on CCL2, CCL7, and Cx3CL1 gene expression could not be found in neither LV- nor SM-derived fibroblasts (**Figures 3A, B**). However, in response to IL-6 stimulation, the secreted CCL7 in the supernatant of cultured LV- and SM-derived fibroblasts was increased (1.3-fold, p = 0.0183) and tended to be higher in LV-derived fibroblasts versus basal conditions (1.2-fold; not significant). In parallel, IL-6 stimulation raised Cx3CL1 by 1.1-fold (p = 0.0306) in the supernatant of LV-derived fibroblasts, whereas no changes were detected in the supernatant of SM-derived fibroblasts (**Figures 3C–E**). CCL2 and CCL7 have been identified as chemokines attracting pro-inflammatory monocytes, whereas Cx3CL1 attracts anti-inflammatory monocytes (35, 36) CCL2/Cx3CL1 and CCL7/Cx3CL1 ratios were calculated. Whereas the CCL2/Cx3CL1 ratio did not differ among the LV and SM groups, the CCL7 to Cx3CL1 ratio was 3.0-fold (p = 0.0196) increased in stimulated SM-derived fibroblasts compared to the respective LV group (**Figures 3F, G**), indicating that—upon IL-6 stimulation—fibroblasts from the SM attract a higher proportion of pro-inflammatory monocytes compared to fibroblasts from the LV.

**Figure 4:**
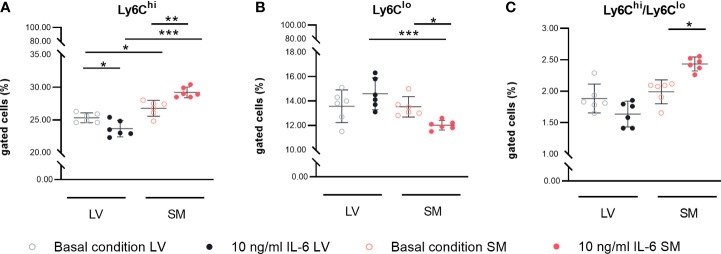
IL-6 stimulation leads to attraction of different monocyte subsets to left ventricle (LV)- versus skeletal muscle (SM)-derived fibroblasts. Percentage of gated **(A)** CD11b^+^CD115^+^Ly6C^hi^ and **(B)** CD11b^+^CD115^+^Ly6C^lo^ monocytes and **(C)** ratio of Ly6C^hi^ versus Ly6C^lo^ monocytes attracted by the medium of LV-derived (black) and SM-derived (red) fibroblasts after 72-h culture with basal medium without IL-6 (basal condition; bright hollow circle) or with 10 ng/ml of IL-6 (dark filled circle), n = 18, N = 3; Kruskal–Wallis/one-way ANOVA. All data are presented as mean ± 95% CI and tested with *post-hoc* Benjamini–Hochberg correction; adjusted p-values: *p < 0.05, **p < 0.01, ***p < 0.001.

Following the observed differences in chemokine expression profile, we further analyzed the potential to attract different monocyte subsets by performing a migration assay. We found a 1.4-fold (p = 0.0197) higher attraction of Ly6C^hi^ cells toward supernatant of unstimulated SM- versus LV-derived fibroblasts. The higher ratio of CCL7/Cx3CL1 in the supernatant of IL-6-stimulated SM-derived versus LV-derived fibroblasts went along with a 2.1-fold (p < 0.0001) higher attraction of Ly6C^hi^ monocytes. Furthermore, IL-6 stimulation led to a 1.3-fold (p = 0.0330) lower attraction of Ly6C^lo^ monocytes by the supernatant of SM-derived fibroblasts compared to basal conditions. Compared to IL-6-stimulated LV-derived fibroblasts, the supernatant of IL-6-stimulated SM-derived fibroblasts resulted in a 2.0-fold (p = 0.0009) reduction in Ly6C^lo^ monocytes. In accordance with this, IL-6-stimulated SM- versus LV-derived fibroblasts IL-6 stimulation led to a 4.3-fold (p = 0.0003) increase in attracted Ly6C^hi^ versus Ly6C^lo^ monocytes by the supernatant of fibroblasts from the SM compared to the LV (**Figures 4A–C**).

### IL-6 stimulation induces NLRP3 inflammasome activity in left ventricle- and skeletal muscle-derived fibroblasts

**Figure 5:**
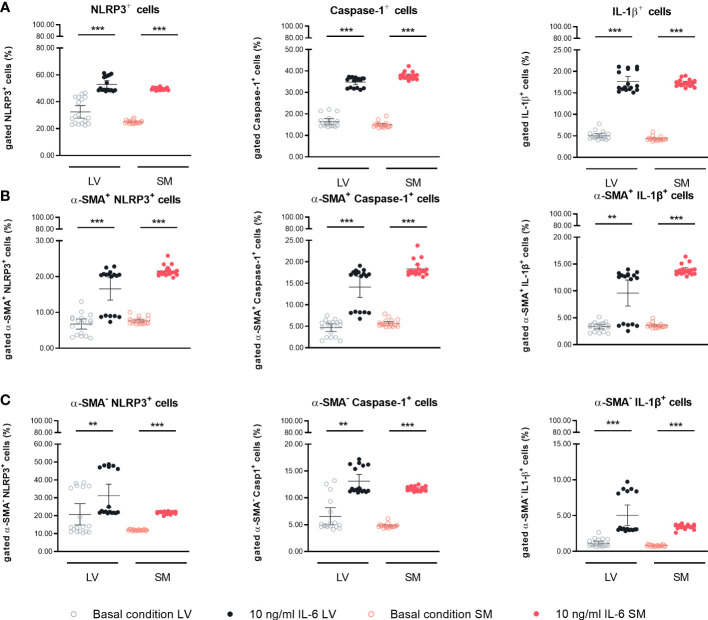
IL-6 stimulation leads to upregulation of NLRP3 inflammasome activity in left ventricle (LV)- and skeletal muscle (SM)-derived fibroblasts. Percentage of gated **(A)** NLRP3^+^, Caspase-1^+^, and IL-1β^+^ cells in the global population; **(B)** NLRP3^+^, Caspase-1^+^, and IL-1β^+^ cells in the α-SMA^+^ subpopulation; and **(C)** NLRP3^+^, Caspase-1^+^, and IL-1β^+^ cells in the α-SMA^−^ subpopulation of LV-derived (black) and SM-derived (red) fibroblasts after 24-h culture with basal medium without IL-6 (basal condition; bright hollow circle) or with 10 ng/ml of IL-6 (dark filled circle), n = 18, N = 3. All data are presented as mean ± 95% CI and tested with *post-hoc* Benjamini–Hochberg correction; adjusted p-values: **p < 0.01,***p < 0.001.

Next, we investigated the potential of LV- and SM-derived fibroblasts to regulate inflammatory processes in the respective tissues in response to IL-6 in more depth. Fibroblasts are the main source of cardiac NLRP3 inflammasome activity (29), whereas the inflammatory potential of SM-derived fibroblasts is still underexplored. Thus, the expression of NLRP3 inflammasome components in LV- and SM-derived fibroblasts was evaluated. Protein expression of NLRP3 was 1.6-fold (p < 0.0001) and 2.0-fold (p < 0.0001) increased as compared to basal conditions in LV- and SM-originated IL-6-stimulated fibroblasts, respectively. Caspase-1 expression was similarly upregulated in stimulated fibroblasts of both tissues (2.1-fold versus 2.5-fold, p = 0.0003 versus p < 0.0001) in LV- versus SM-derived fibroblasts compared to unstimulated cells. In accordance with the previous results, the expression of the end product of the inflammasome formation, IL-1β, was enhanced in stimulated LV- (3.5-fold, p < 0.0001) and SM-derived fibroblasts (4.0-fold, p < 0.0001) compared to respective unstimulated controls. No difference in NLRP3 inflammasome activity was observed between LV- and SM-derived fibroblasts, under neither basal conditions nor IL-6 stimulation (**Figure 5A**). Following this observation, we further analyzed whether there was a difference in the expression of inflammasome components depending on the α-SMA^+^ versus α-SMA^−^ fibroblast population. Independent of the expression of α-SMA, the NLRP3 inflammasome activity was increased in LV- and SM-derived fibroblasts upon stimulation with IL-6, and no differences among the fibroblasts of the different tissues were observed (**Figures 5B, C**).

### Left ventricle- and skeletal muscle-derived fibroblasts have a differentially regulated mitochondrial and glycolytic metabolism

**Figure 6:**
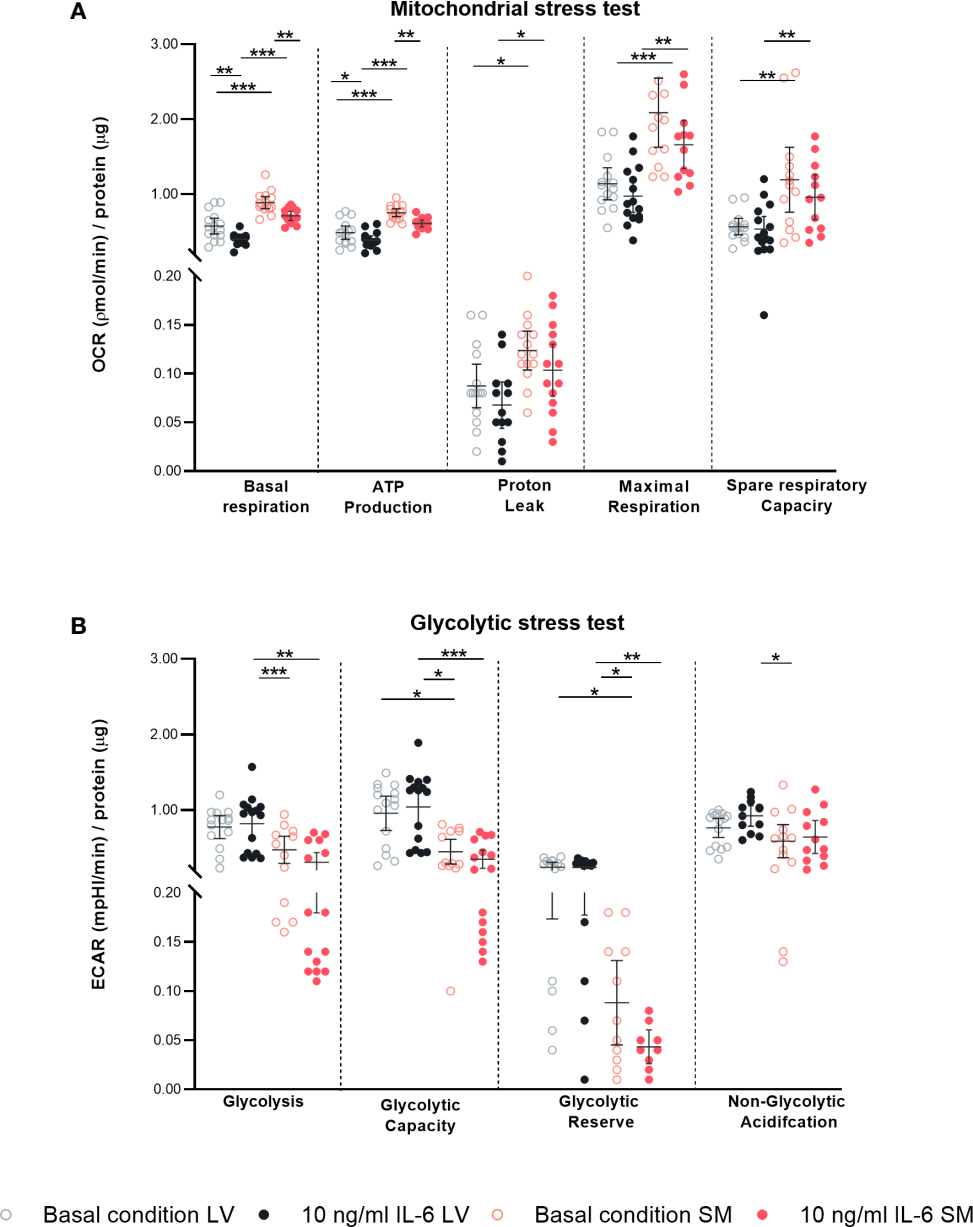
IL-6 stimulation leads to differential regulated mitochondrial and glycolytic disturbances in left ventricle (LV)- versus skeletal muscle (SM)-derived fibroblasts. Mitochondrial stress test measuring **(A)** oxygen consumption rate (OCR) describing basal respiration, ATP production, proton leak, maximal respiration, and spare respiratory and glycolytic stress test assessing **(B)** extracellular acidification rate (ECAR) describing glycolysis, glycolytic capacity, glycolytic reserve, and non-glycolytic acidification normalized to protein amount in μg of LV-derived (black) and SM-derived (red) fibroblasts after 4-h culture with basal medium without IL-6 (basal condition; bright hollow circle) or with 10 ng/ml of IL-6 (dark filled circle); n = 15, N = 3. All data are presented as mean ± 95% CI and tested with *post-hoc* Benjamini–Hochberg correction; adjusted p-values: * p < 0.05, ** p < 0.01, *** p < 0.001.

Since increased IL-6 is strongly associated with disturbances in metabolism in both the heart and SM (37–40) and since inflammation and cellular metabolism are linked *via* NLRP3 inflammasome activity (41, 42), we next investigated the impact of IL-6 on the fibroblast’s mitochondrial function and glycolytic function using a Seahorse assay. Under basal conditions, fibroblasts from the SM showed a higher mitochondrial energy consumption characterized by increased basal respiration (1.6-fold, p < 0.0001), ATP production (1.6-fold, p < 0.0001), proton leakage (1.4-fold, p = 0.0498), maximal respiration (1.8-fold, p < 0.0001), and spare respiratory capacity (2.1-fold, p = 0.0301) as compared to LV-derived fibroblasts. In response to IL-6, LV- and SM-derived fibroblasts showed a decreased mitochondrial metabolic activity indicated by reduced basal respiration (1.5-fold versus 1.3-fold, both p = 0.0014), ATP production (1.3-fold versus 1.2-fold, p = 0.0206 versus p = 0.0024) compared to basal conditions. Both proton leakage and spare respiratory capacity were not regulated upon cytokine stimulation with IL-6, in neither LV- nor SM-derived fibroblasts (**Figure 6A**). We next showed that LV-derived fibroblasts have an increased glycolytic metabolism as compared to fibroblasts from the SM. The glucose-induced overall glycolysis tended to be higher (1.6-fold; not significant), and oligomycin-induced glycolysis (glycolytic capacity) was increased in LV- versus SM-derived fibroblasts (2.1-fold, p = 0.0246). The competence of the LV-derived fibroblasts to adapt to the energy demand (glycolytic reserve; 2-DG-induced glycolysis) was 2.7-fold (p = 0.0087) higher under basal conditions compared to fibroblasts of the SM, further indicating that LV fibroblasts rather rely on glycolytic energy production than SM-derived fibroblasts. IL-6 stimulation did not affect glycolytic function in neither LV- nor SM-derived fibroblasts. Likewise, IL-6 stimulation did not alter other sources of extracellular acidification (non-glycolytic acidification) (**Figure 6B**). Thus, IL-6 induces a metabolic shift toward reduced mitochondrial energy production rather than altered glycolysis in LV- and SM-derived fibroblasts.

### Secretion of gp130 receptor in left ventricle- versus skeletal muscle-derived fibroblasts

**Figure 7:**
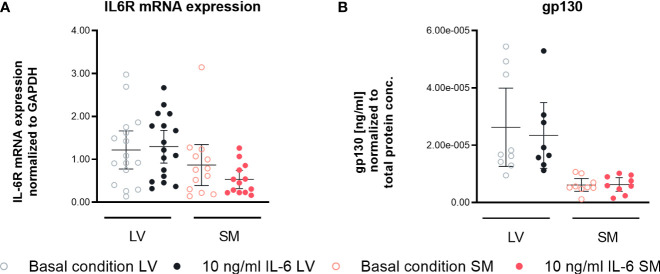
IL-6 *trans*-signaling is differently regulated in left ventricle (LV)- versus skeletal muscle (SM)-derived fibroblasts. **(A)** IL6R mRNA expression normalized to GAPDH and respective control of LV-derived (black) and SM-derived (red) fibroblasts after 24-h culture with basal medium without IL-6 (basal condition; bright hollow circle) or with 10 ng/ml of IL-6 (dark filled circle), n = 18, N = 3, Kruskal–Wallis/one-way ANOVA. **(B)** Protein expression of gp130 in the supernatant normalized to total protein content measured *via* bicinchoninic acid assay of LV-derived (black) and SM-derived (red) fibroblasts after 72-h culture with basal medium without IL-6 (basal condition; bright hollow circle) or with 10 ng/ml of IL-6 (dark filled circle). All data are presented as mean ± 95% CI and tested with *post-hoc* Benjamini–Hochberg correction.

To obtain insights into the different biological responses in fibroblasts from the LV and SM upon IL-6 stimulation, we analyzed the expression of the IL-6R and gp130. According to our data, IL-6 stimulation did not affect IL-6R expression on mRNA level. However, secretion of the IL-6 co-receptor gp130 tended to be higher in unstimulated (4.3-fold, not significant) and stimulated LV-derived fibroblasts (3.7-fold, not significant) compared to fibroblasts from the SM (**Figure 7**), suggesting a different potential in the regulation of IL-6 *trans*-signaling between LV- and SM-derived fibroblasts.

## Discussion

In this study, we showed that LV- and SM-derived fibroblasts differ in basal expression of ECM components and chemokines. IL-6 stimulation led to the induction of an α-SMA-expressing phenotype, increased NLRP3 inflammasome activity, and de-regulated mitochondrial metabolism in both tissue fibroblasts, by which LV-derived fibroblasts are characterized with a chemokines profile attracting anti-inflammatory monocytes, whereas SM-derived fibroblasts attract pro-inflammatory monocytes.

Single-cell analysis of fibroblasts from the heart and SM indicates different fibroblast phenotypes and an organ-specific fibroblast-mediated ECM profile (28). Biologically, intra-organ heterogeneity of fibroblasts on a basal level may be related to the diverse microenvironment of the fibroblasts in different tissues, including different surrounding cells, different mechanosensation, and metabolism. Indeed, the LV and quadriceps consist of two different types of muscles (cardiomyocytes versus SM cells) exhibiting a location-dependent physiological environment and cellular demand. In agreement, we show that LV- and SM-derived fibroblasts differ in their basal expression of ECM components and chemokines by which fibroblasts from the SM have a higher expression, except for basal LOXL2 expression, which is reduced compared to fibroblasts from the LV. In addition, while LV-derived fibroblasts seem to be more dependent on glucose metabolism compared to SM-derived fibroblasts, the mitochondrial activity is higher in unstimulated SM- compared to LV-derived fibroblasts.

We next investigated whether LV- and SM-specific fibroblasts differ in their responsiveness to IL-6, analyzing potential distinct biological functions of fibroblasts at different local sites. Stimulation of LV- and SM-originated fibroblasts with IL-6 led to transdifferentiation of fibroblasts from both tissues into an α-SMA-expressing phenotype. In addition to producing and modulating ECM, activated (myo)fibroblasts are well known to strongly influence cardiac inflammation (29, 34). A central function of cardiac fibroblasts is the attraction of immune cells *via* chemokine expression (34). The comparison of chemokine expression from fibroblasts of both tissues did not show any difference in CCL2 expression between LV- and SM-derived fibroblasts supplemented with IL-6. In contrast, CCL7 expression was increased in IL-6-stimulated LV- and SM-derived fibroblasts, whereas CX3CL1 was only upregulated following IL-6 stimulation in LV fibroblasts. CCL7 is a pro-inflammatory regulator of cardiac remodeling and critical for the recruitment of monocyte subsets in the myocardium (43, 44). CX3CL1 functions in a later phase of the myocardial healing process by mediating the attraction of anti-inflammatory monocytes and subsequent support of tissue repair by modulating the aggregation of activated cardiac (myo)fibroblasts (35). Little is known about the impact of fibroblasts in the SM on inflammation and notably about the influence on the induction of tissue-specific immune responses. Here, we show that IL-6 stimulation increased the release of CCL7 to CX3CL1 in the supernatant of SM-originated fibroblasts, hinting to a possible role of SM-derived fibroblasts driving a pro-inflammatory immune response. This is supported by our migration assay data, illustrating the attraction of more pro-inflammatory Ly6C^hi^ than anti-inflammatory Ly6C^lo^ monocytes versus supernatant of IL-6-stimulated SM fibroblasts. In addition, IL-6 increased NLRP3 inflammasome activity in SM fibroblasts. These findings corroborate the contribution of SM fibroblasts in the less recognized presence of muscle inflammation (8, 9) and pro-inflammatory monocyte presence (10, 45) in muscles of patients with HF and circulating cytokines. Instead, fibroblasts from the LV potentially play an ambivalent role in driving inflammation following IL-6 by, on the one hand, supporting anti-inflammatory immune responses attracting Ly6C^lo^ monocytes and, on the other hand, exhibiting high NLRP3 inflammasome activity, thus supporting pro-inflammatory reactions upon IL-6 stimulation. It further accentuates how LV fibroblasts may act differently on the inflammatory response as previously already shown for TGF-β and IFN-γ (22).

Differences in the responsiveness of LV- and SM-derived fibroblasts to stimulation with IL-6 might be explained by variations in the fibroblast’s IL-6R expression (46, 47), involving signaling over membrane-bound IL-6R (via *cis* signaling). Following our findings, IL-6R expression did not differ among LV- and SM-derived fibroblasts, although we observed a higher secretion of the natural antagonist of the soluble IL-6R, soluble gp130 receptor (48, 49) by LV- compared to SM-derived fibroblasts. Fibroblasts also respond to *trans* IL-6 signaling (47), involving the binding of a IL-6/IL-6R complex to the ubiquitously expressed gp130 receptor (50). Thus, differences in *trans* IL-6 signaling in LV- and SM-derived fibroblasts might be a reason for the diverse response to IL-6. The higher secretion of the natural antagonist of the soluble IL-6R, soluble gp130 receptor (48, 49), by LV- compared to SM- fibroblasts hints at a function of LV-derived fibroblasts in protecting the heart from detrimental systemic effects of IL-6, which is also supported by the chemokine profile in stimulated cells. IL-6 is known to be the first protective during acute cardiac inflammation, but excessive IL-6 during chronic inflammation leads to malignant effects on cardiac function (16, 51). Here, LV-derived fibroblasts might play an important role in mediating the balance between cardio-protection and chronic inflammatory response in the heart. IL-6 induces NLRP3 inflammasome activity and IL-1β production in macrophages (52). IL-1β further drives the production of IL-6, creating a positive feedback loop in fibroblasts (53), which might, due to an excess of local IL-6, lead to a shift in the fibroblast’s phenotype in the heart in the course of systemic inflammation.

In the SM instead, the effect of IL-6 on the fibroblasts rather implicates direct regulation of the immune reaction to pro-inflammatory responses, further demonstrating the location-dependent biological function of fibroblasts from different organs in the context of inflammation. In the SM, it has been shown that IL-6 induced aberrant mitochondrial metabolism (37). This further draws attention to the role of the NLRP3 inflammasome activity in linking metabolism and inflammation not only in the heart (42) but in the muscle as well. The NLRP3 inflammasome is known to become activated upon metabolic disturbances and boosts pro-inflammatory immune reactions *via* IL-1β, which not only drives myocardial remodeling (42) but also is the hallmark of cachexia, metabolic de-regulation (54, 55). According to the data presented here, IL-6 could be here an important mediator between de-regulated metabolism in cachexia and inflammatory response in fibroblasts from the LV and SM regarding NLRP3 inflammasome activity. Furthermore, on a cellular level, analysis of mitochondrial function revealed that treatment with IL-6 leads to a significant reduction of basal and ATP-linked respiration in fibroblasts from both tissues, indicating either deregulation of ATP use or synthesis of substrates being used for oxidization. In a physiological state, the heart highly depends on the generation of ATP *via* oxidative phosphorylation in the mitochondria (>95%) and only to a less extent *via* glycolysis (56). In the SM, fast switches from glycolytic to mitochondrial metabolism allow to metabolically adapt to short-term and sustained activities (57). Disturbances in mitochondrial energy production have been reported in both SM cells and the cardiomyocytes of cachectic mice (19, 58, 59). As a response to disturbed mitochondrial metabolism in HF, a switch to a higher contribution of glycolysis to overall energy production is observed (60). Therefore, we performed a glycolytic stress test to further understand the shifted metabolic processes in IL-6-stimulated fibroblasts. Here, we showed that IL-6 did not affect glycolysis in LV-derived fibroblasts, whereas glycolysis, glycolytic capacity, and reserve tended to be lower in SM fibroblasts following IL-6 stimulation, suggesting a higher sensitivity to change in glycolysis in SM fibroblasts, although with respect to both tissues, IL-6 mainly regulates mitochondrial metabolism, which can further drive the fibroblast’s inflammatory phenotype. This highlights that the NLRP3 inflammasome might be directly regulated either by IL-6 or *via* disturbances in mitochondrial rather than in glycolytic metabolism in fibroblasts from the LV and SM.

IL-6 is a mediator of systemic inflammatory responses not only in cancer and HF but also in other inflammatory and infectious diseases. For instance, a link between cardiovascular diseases and SARS-CoV-2 infection is represented by high IL-6 levels in patients with severe disease progression. IL-6 is here discussed not only to be a biomarker describing the severity and outcome of the disease but also to play a role as a potential therapeutic target against COVID-19 (61).

Based on our findings illustrating that IL-6 induces NLRP3 inflammasome activity and hence the inflammatory potential of fibroblasts in the SM and LV, fibroblasts might be able to serve as positive feedback amplifiers of local and subsequent systemic IL-1β and IL-6 production, which would lead to severe functional effects in the heart (5). Beneficial effects are shown following NLRP3-, IL-1β-, and IL-6-inhibiting drugs on HF (62, 63), which might therefore be partly explained by the inhibition of the pro-inflammatory potential of LV and SM fibroblasts.

We conclude that SM- and LV-derived fibroblasts differ under basal conditions and following IL-6 supplementation. Their differential responsiveness to IL-6 in terms of attraction of different monocyte subclasses suggests a different contribution of SM and LV fibroblasts to local and (potential) systemic inflammation in cancer, HF, and hereto related cachexia. However, the similar responsiveness of SM and LV fibroblasts related to NLRP3 activation following IL-6 suggests that SM-derived fibroblasts could be potentially used as a tool to mirror the cardiac inflammatory NLRP3 response during cancer progression prior to cardiac damage.

## Data availability statement

The original contributions presented in the study are included in the article/**supplementary material**. Further inquiries can be directed to the corresponding author.

## Ethics statement

The animal study was reviewed and approved by Landesamt für Gesundheit und Soziales, Berlin (LAGeSo), Turmstraße 21, Haus A, 10559 Berlin.

## Author contributions

IM wrote the manuscript and substantially contributed to the study design, data acquisition, data analysis, and data interpretation. KP substantially contributed to the cell culture of the cardiac and SM fibroblasts and subsequent molecular investigations. JS contributed to the study design and revised the manuscript. SVL conceived the study, provided funding, contributed to the data analysis and data interpretation, and revised the manuscript. All authors revised the manuscript for intellectual content and gave their final approval for publication.

## Funding

The project was supported by the DFG, SFB 1470 to SL.

## Acknowledgments

We thank Annika Koschel, Kerstin Puhl, and Marzena Sosnowski (in alphabetical order) for excellent technical support. Furthermore, we thank Alba del Rio Serrato (AG Infante-Duarte, Experimental and Clinical Research Centre, Max-Delbrück Centre (MDC) and Charité Universitätmedizin Berlin, Berlin) for kindly providing the bEnd.3 cells.

## Conflict of interest

The authors declare that the research was conducted in the absence of any commercial or financial relationships that could be construed as a potential conflict of interest.

## Publisher’s note

All claims expressed in this article are solely those of the authors and do not necessarily represent those of their affiliated organizations, or those of the publisher, the editors and the reviewers. Any product that may be evaluated in this article, or claim that may be made by its manufacturer, is not guaranteed or endorsed by the publisher.
