# Supplementary material for: Left ventricle- and skeletal muscle-derived fibroblasts exhibit a differential inflammatory and metabolic responsiveness to interleukin-6
Source: Front Immunol. 2022 Jul 28;13:947267. doi: 10.3389/fimmu.2022.947267 (PMC9366145; doi:10.3389/fimmu.2022.947267)
Supplement: Supplementary file 1 [file DataSheet_1.docx]

Supplementary Material

# Supplemental Figures


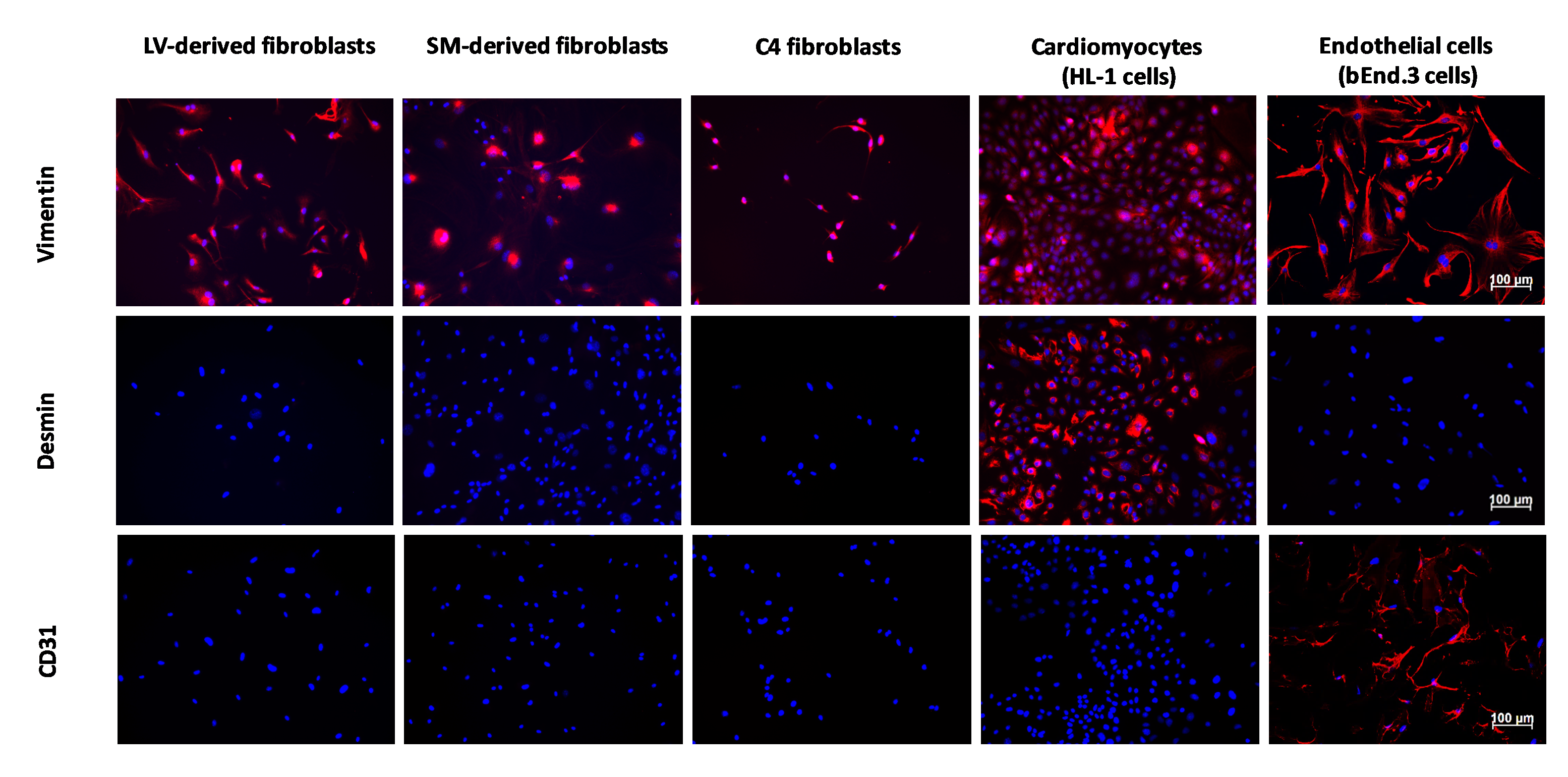


**Supplemental Figure 1.** **Characterization of primary tissue-derived fibroblasts from left ventricle and skeletal muscle.** Panel illustrates vimentin, desmin, and CD31 marked by a Cy3-conjugated streptavidin antibody (red) and DAPI for nuclear staining (blue) in primary LV- and SM-derived fibroblasts cultures (passage 1) generated via outgrowth culture, a fibroblast cell line (C4 cells), cardiomyocytes (HL-1 cells) and brain-derived endothelial cells (bEnd.3 cells) (from left to right). Imaging was performed at 200x magnification (scale bar = 100 µm).


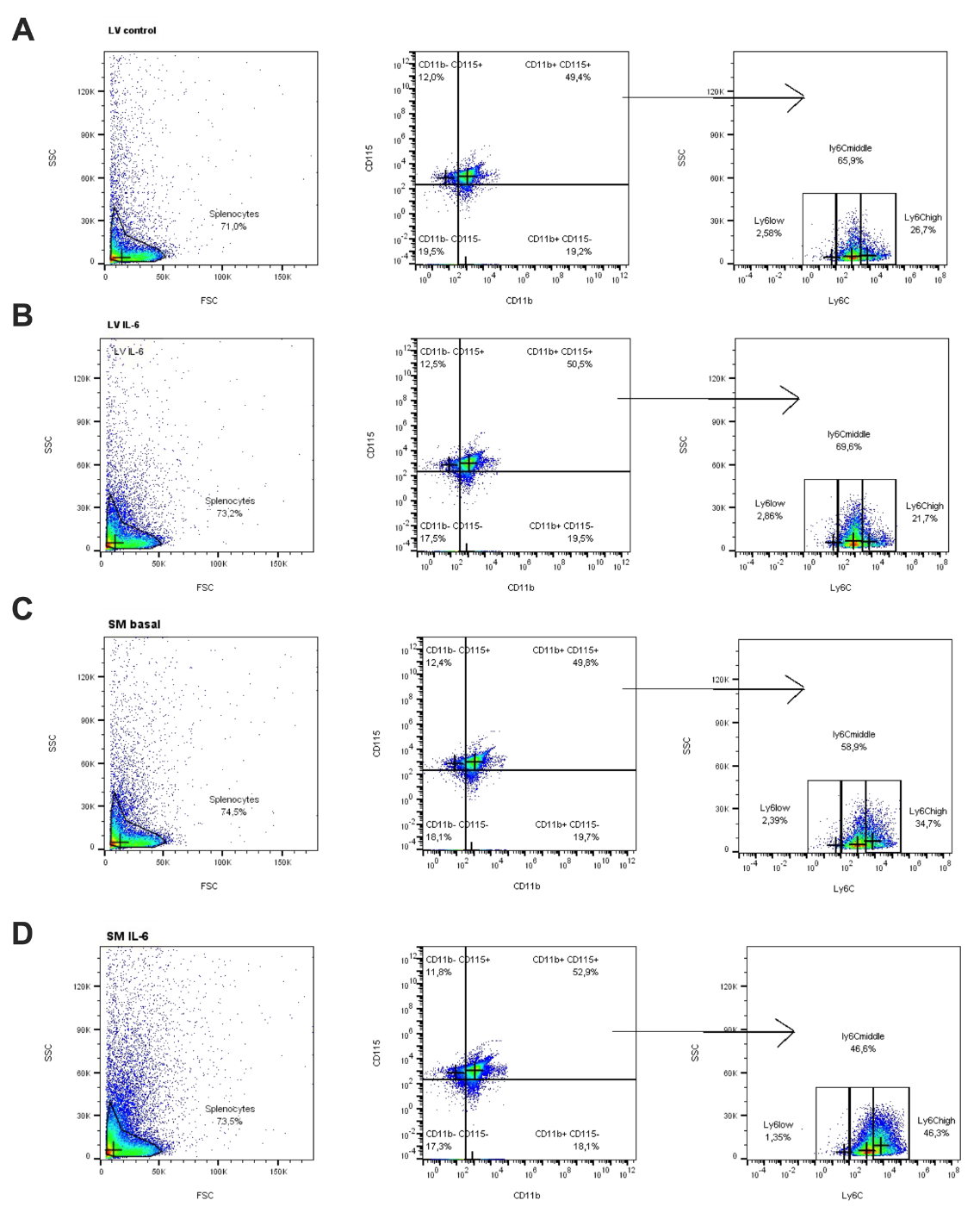


**Supplemental Figure 2. Flow cytometry gating strategy to identify migrated splenocyte subpopulations.** Flow cytometry gating strategy to identify CD115^+^CD11b^+^Ly6C^+^ populations of splenocytes migrated towards the supernatant of unstimulated and stimulated LV- (A-B) and SM- derived fibroblasts (C-D) after 72 h stimulation with IL-6. Splenocytes were gated based on SSC-A versus FSC-A. Ly6C^lo^, Ly6C^mid^ and Ly6C^hi^ cells were selected from the CD115^+^ and CD11b^+^ gate.


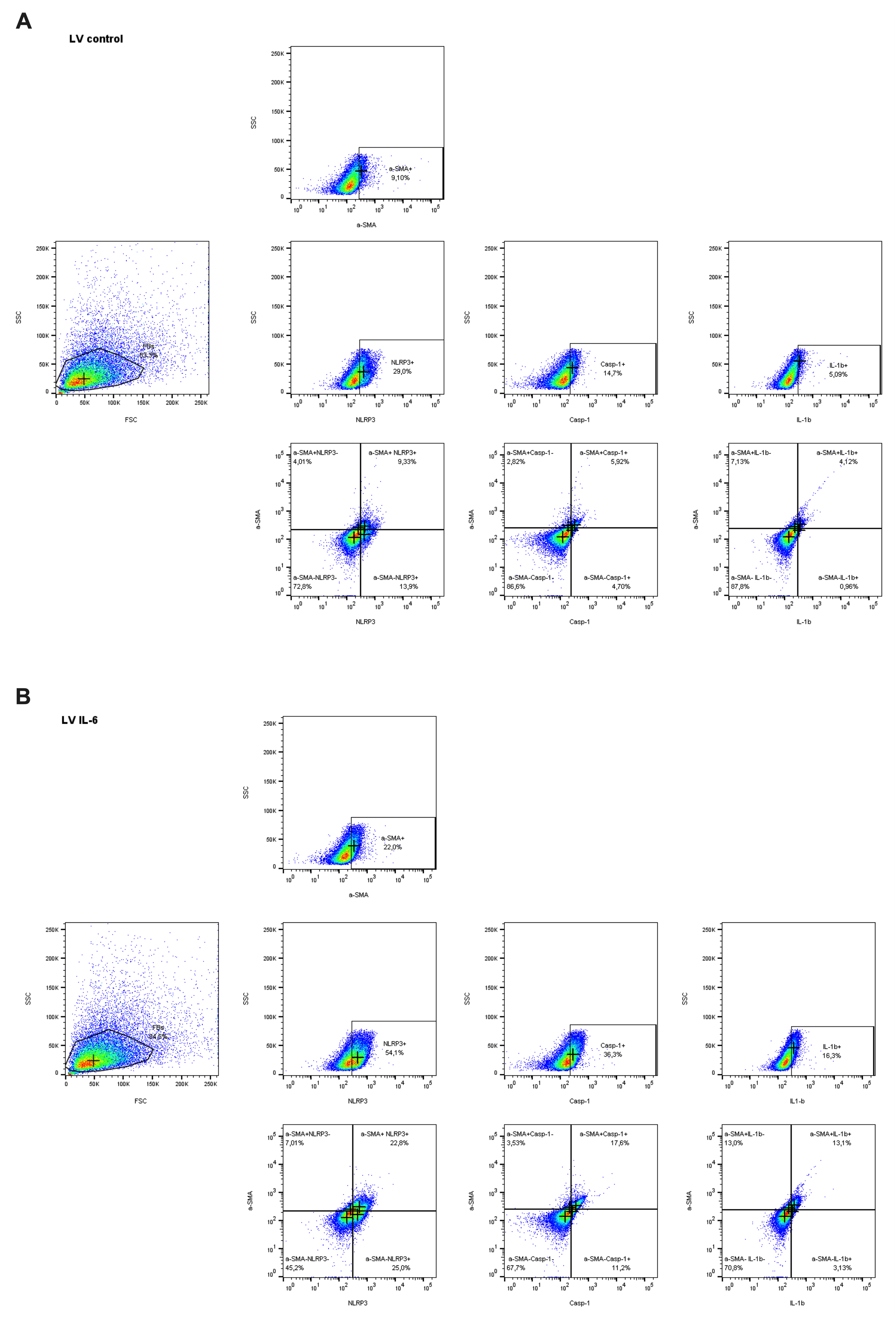


**Supplemental Figure 3.** **Flow cytometry gating strategy to identify** **NLRP3 inflammasome activity in LV-derived fibroblasts**. Flow cytometry gating strategy to identify α-SMA^+^, NLRP3^+^, Caspase-1^+^, IL-1β^+^, α-SMA^+^ NLRP3^+^, α-SMA^+^ Caspase-1^+^, α-SMA^+^ IL-1β^+^, α-SMA^-^ NLRP3^+^, α-SMA^-^ Caspase-1^+^, α-SMA- IL-1β^+^ populations of unstimulated (A) and stimulated (B) LV-derived fibroblasts after 24 h stimulation with IL-6. Fibroblasts were gated based on SSC-A versus FSC-A from which α-SMA^+^, NLRP3^+^, Caspase-1^+^, IL-1β^+^, α- SMA^+^ NLRP3^+^, α-SMA^+^ Caspase-1^+^, α-SMA^+^ IL-1β^+^, α-SMA^-^ NLRP3^+^, α-SMA^-^ Caspase-1^+^, α-SMA^-^ IL-1β^+^ were selected.


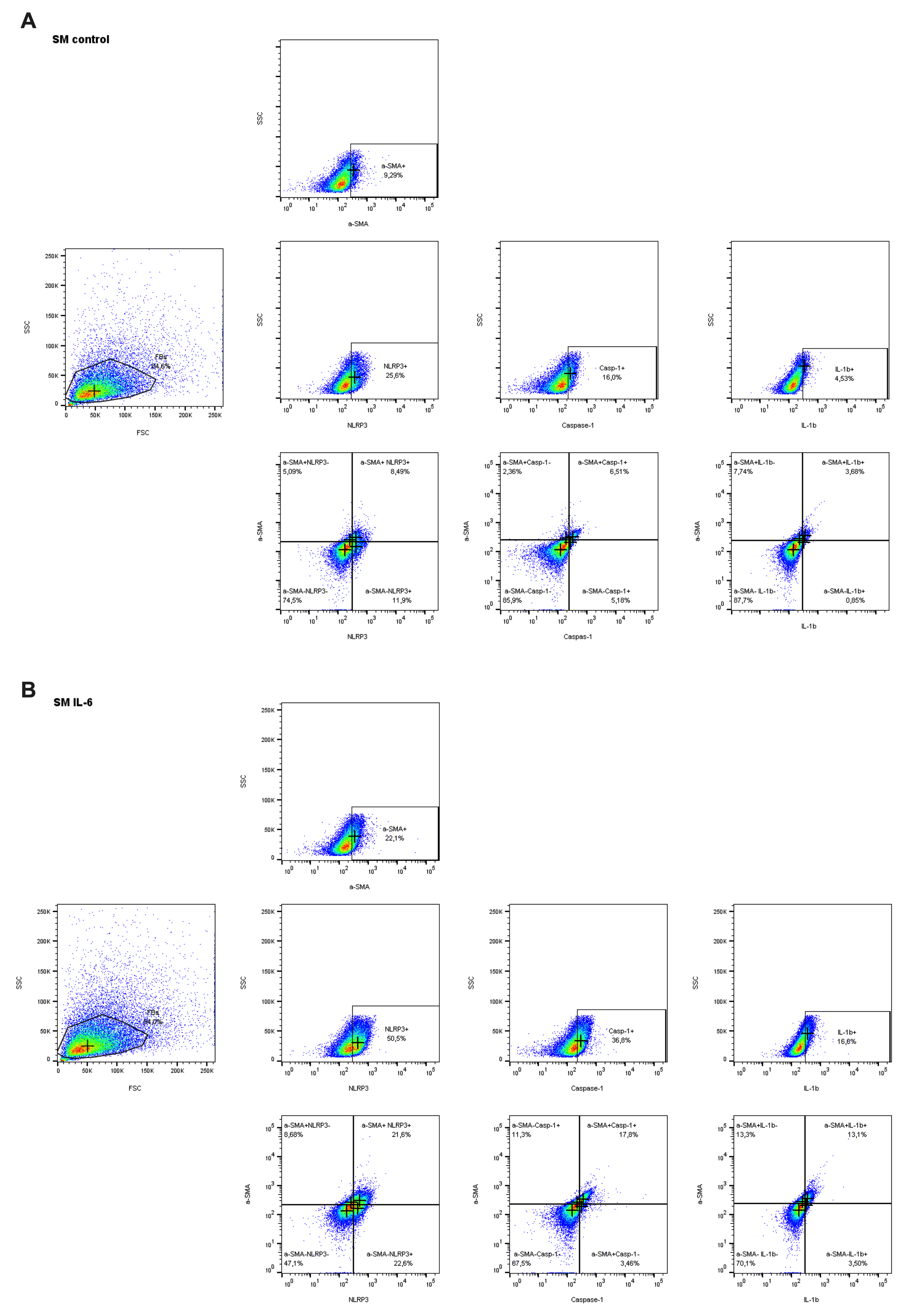


**Supplemental Figure 4.** **Flow cytometry gating strategy to identify** **NLRP3 inflammasome activity in SM-derived fibroblasts.** Flow cytometry gating strategy to identify α-SMA^+^, NLRP3^+^, Caspase-1^+^, IL-1β^+^, α- SMA^+^ NLRP3^+^, α-SMA^+^ Caspase-1^+^, α-SMA^+^ IL-1β^+^, α-SMA- NLRP3^+^, α-SMA^-^ Caspase-1^+^, α-SMA^-^ IL-1β^+^ populations of unstimulated (A) and stimulated (B) SM-derived fibroblasts after 24 h stimulation with IL-6. Fibroblasts were gated based on SSC-A versus FSC-A from which α-SMA^+^, NLRP3^+^, Caspase-1^+^, IL-1β^+^, α- SMA^+^ NLRP3^+^, α-SMA^+^ Caspase-1^+^, α-SMA^+^ IL-1β^+^, α-SMA^-^ NLRP3^+^, α-SMA^-^ Caspase-1^+^, α-SMA^-^ IL-1β^+^ were selected.
